# Axiom, Anguish, and Amazement: How Autistic Traits Modulate Emotional Mental Imagery

**DOI:** 10.3389/fpsyg.2016.00757

**Published:** 2016-05-30

**Authors:** Gianluca Esposito, Sara Dellantonio, Claudio Mulatti, Remo Job

**Affiliations:** ^1^Department of Psychology and Cognitive Science, University of TrentoRovereto, Italy; ^2^Division of Psychology, Nanyang Technological UniversitySingapore, Singapore; ^3^Department of Developmental Psychology and Socialization, University of PaduaPadua, Italy

**Keywords:** mental imagery, autistic traits, emotion recognition, emotion regulation, proprioception

## Abstract

Individuals differ in their ability to feel their own and others’ internal states, with those that have more autistic and less empathic traits clustering at the clinical end of the spectrum. However, when we consider semantic competence, this group could compensate with a higher capacity to imagine the meaning of words referring to emotions. This is indeed what we found when we asked people with different levels of autistic and empathic traits to rate the degree of imageability of various kinds of words. But this was not the whole story. Individuals with marked autistic traits demonstrated outstanding ability to imagine theoretical concepts, i.e., concepts that are commonly grasped linguistically through their definitions. This distinctive characteristic was so pronounced that, using tree-based predictive models, it was possible to accurately predict participants’ inclination to manifest autistic traits, as well as their adherence to autistic profiles – including whether they fell above or below the diagnostic threshold – from their imageability ratings. We speculate that this quasi-perceptual ability to imagine theoretical concepts represents a specific cognitive pattern that, while hindering social interaction, may favor problem solving in abstract, non-socially related tasks. This would allow people with marked autistic traits to make use of perceptual, possibly visuo-spatial, information for “higher” cognitive processing.

## Introduction

The imageability scale is a classical psycholinguistic measure for assessing the degree to which words evoke mental images (Paivio et al., 1968; Connell and Lynott, 2012). Mental images (or imageries) are non-verbal re-presentations (i.e., re-calls) of any kind of sensory experience we were exposed to in the past (Roeckelein, 2004). They are associated with words, reflect the strength of the link between specific words and sensory experiences, and have behavioral consequences as high-imageability items are more easily processed and understood than low-imageability items (Paivio, 1971, 1986, 1991, 2007). In this study we want to assess how this specific psycholinguistic construct interacts with individuals’ ability to understand their own and others’ internal states. Specifically, we want to explore whether the imageability ratings of specific word categories predict an individual’s autistic or empathic traits, measuring levels of autistic and empathy traits along a continuum in which all of us lie that goes from very low to very high (on the link between empathy and autism see e.g., Dalsant et al., 2015; Gentili et al., 2015).

Usually the link between imagery and words is investigated in relation to the external senses. Studies on imagery show, however, that we form mental images of all kinds of sensory experiences, including motor, kinaesthetic proprioceptive, interoceptive, and emotional experiences (see e.g., Switras, 1978; Gollnisch and Averill, 1993; Acerra and Moseley, 2005; Anema and Dijkerman, 2013). In line with these results, in previous research we showed that the imageability scale developed by Paivio captures not only the degree of imageability of words that denote things that can be perceived by the external senses, but also that of words that denote internal states, specifically those referring to emotive, proprioceptive, and interoceptive states (Dellantonio et al., 2014a,b; Pastore et al., 2015).

As individuals differ in their ability to recognize their own and others’ internal states, with those with higher levels of autistic traits and lower empathy traits clustering at the lower end of the spectrum (Ronald et al., 2006), people belonging to this group could compensate for this gap by being more able than others to form mental images associated with emotive, proprioceptive, and interoceptive words^1^. Since the imageability scale also assesses the degree to which words referring to internal states evoke mental images, the imageability rating of word classes denoting these states could allow us to predict whether people have low or high autistic traits, or, in other words, whether they have more or less marked empathic inclinations.

### Autistic Traits, Imageability, and Word Classes

The main tools to detect individuals’ levels of autistic traits are two self-report questionnaires developed by Baron-Cohen et al. (2001), the Autism Quotient and the Empathy Quotient. A person’s Autism Quotient score represents the quantity of Autism Spectrum Disorder (ASD) traits s/he shows in her/his behavior, whereas the Empathy Quotient measures empathy traits related to the recognition of others’ emotions and moods, the lack of which is involved in ASD impairments. As is already suggested by the link between autism and empathy, people with marked autistic traits are less able than others to identify internal states. Specifically, they have difficulties recognizing both their own and others’ emotions and introspective feelings (Gaigg, 2012; Matsuda and Yamamoto, 2015). For this reason, one would expect them to also have a lower capacity to imagine (i.e., to dynamically form and mentally re-evoke) emotional and introspective experiences.

The aim of this study is to test whether autistic traits correlate with the capacity to imagine different kinds of words and thus whether the imageability scale, and specifically the ratings obtained by it (Paivio et al., 1968; Coltheart, 1981; Wilson, 1988; Connell and Lynott, 2012), can be used as a means to predict how many autistic or, at the other end of the continuum, empathic traits a person possesses. A correlation of this kind would not only offer a new and unexpected psycholinguistic instrument to assess whether people are characterized by varying degrees of autistic traits, but it would also provide important cues concerning the cognitive ‘styles’ and the semantic competence of persons with more or less marked autistic traits. Specifically, it might allow us to understand whether and in which cases people with varying degrees of autistic traits tend to strongly associate words with perceptual representations.

Different words refer to concepts that rely to different degrees on three main kinds of information: external sensory information (e.g., ‘table,’ ‘fennel,’ and ‘candle’), internal sensory information (i.e., proprioceptive and emotional information, e.g., ‘pain,’ ‘hunger,’ ‘fever,’ and ‘cramp’ as well as ‘happiness,’ ‘anger,’ and ‘disgust’) and linguistic information (e.g., ‘fallacy,’ hypothesis,’ ‘axiom,’ ‘fraud,’ and ‘democracy’; Barsalou, 1999, 2008; Prinz, 2002; Barsalou et al., 2003; Barsalou and Wiemer-Hastings, 2005; Vigliocco et al., 2009; Kousta et al., 2011). Imagery is a measure of the link between words and sensory information, therefore words with high imagery ratings will be more strongly linked with perception (either internal or external), while words with low imagery ratings will rely more heavily on linguistic information and be understood mainly on the basis of linguistic definitions: they will be more abstract or, more precisely, theoretical (‘abstract’ is often used for describing ‘non-material’ objects; in this sense, words denoting emotions or feelings would also be abstract). On the basis of this analysis we identified four word classes: the first two include words referring to internal states, i.e., specifically (i) proprioceptive and (ii) emotional states; (iii) the third consists of theoretical words and (iv) the last consists uniquely of *concrete* nouns; this last group also serves as a control since it is matched with the others for frequency and length.

Since people with autism have a limited capacity to recognize internal feelings, we expect that they will be less able to imagine words referring to emotional and proprioceptive states and that therefore imageability ratings assigned to these word classes will negatively correlate with autistic traits. On the other hand, there is no evidence that more or less marked autistic traits will influence the way people perceive the external world or the way they grasp verbal definitions. Thus, imageability ratings of concrete and of theoretical words should remain stable independently of the degree of autistic traits. To assess this hypothesis, we tested whether the imageability ratings assigned to the four classes of words correlates with the degree of participants’ autistic/empathic traits as calculated by the Autism Quotient and the Empathy Quotient.

### Aim of the Study

This study aims at investigating whether more or less marked autistic and/or empathic traits modulate semantic competence with specific word classes. We hypothesize that individuals with higher autistic traits and lower empathic skills have a less developed capacity to imagine the meaning of words referring to emotions and proprioceptive feelings. To test this hypothesis, imageability judgements were submitted to recursive partitioning (also known as tree-based models; Costello et al., 2003) to analyze how the words’ category (theoretical, emotional, proprioceptive, and concrete) predicts the amount of autistic (or empathy) traits. Given a set of independent variables (word category) and a dependent variable (level of autistic or empathy traits), tree-based models provide unique information about the (1) hierarchy of contributions from independent variables in explaining the distribution of the dependent variable and (2) which value of the independent variable subdivides the dependent variable into statistically different groups. To evaluate whether the results were broadly general or clinically specific, we selected adults along a continuum, from low to high autistic traits.

## Materials and Methods

### Participants

One-hundred and seventy-one participants aged between 18 and 58 years (*N* = 171; *M* = 31.6 years; *SD* = 10.9 years), 104 female (*M* = 30.62 years; *SD* = 10.8 years), 67 male (*M* = 33.1 year; *SD* = 10.9 years) participated in the study. Their educational level was medium-high (with all the participants having at least completed high school). Participants were recruited through the Department of Psychology and Cognitive Science electronic notice board. Furthermore, to increase variability in the level of autistic/empathy traits, invitation to participate in the study was electronically sent to an association of people with Asperger Syndrome. Although we were able to differentiate among the participants that were recruited from the Asperger Syndrome association (*N* = 62; 31 m/31f) from those that were recruited from the general population (*N* = 109; 36 m/73f), we were not able to confirm their clinical diagnosis. For these reasons we grouped all the participants together assuming a continuous distribution of autistic and empathy traits between clinical and sub-clinical (normal) populations. We used the Autism Quotient Questionnaire (AQ) and the Empathy Questionnaire (EQ) to assess each participant’s location along the spectrum of Autistic/Empathy traits. Participants recruited from the Asperger Syndrome association had significantly [*F*(1,169) = 151.1, *p* < 0.001] higher levels of AQ (*M* = 35.4; *SD* = 7.3) than participants from the subclinical group (*M* = 18.3; *SD* = 9.5) and had significantly [*F*(1,169) = 125.7, *p* < 0.001] lower levels of EQ (*M* = 21.8; *SD* = 10.2) than participants from the subclinical group (*M* = 43.3; *SD* = 12.9). However, using the Kolmogorov–Smirnov test we confirmed the normality of our AQ (*D* = 0.11, *p* = 0.24) and EQ (*D* = 0.11, *p* = 0.19) score distributions. In spite of this, we cannot exclude the possibility that the extreme group of participants with a potential diagnosis of Asperger Syndrome might have had a relatively strong effect on the final results.

Participants were provided with information on the basic background of the study and the procedure before giving their consent. Participants did not receive any monetary incentive to participate in the study. The study was approved by the Internal Ethical Committee of the University of Padova, and was conducted according to the principles expressed in the Declaration of Helsinki. Informed consent was obtained from the participants.

### Materials

#### Words

One hundred and eighty Italian words were selected as experimental stimuli. Each word fell in one of three categories (*N* = 60): emotional words, proprioceptive words, theoretical words. In addition, 180 words were selected as control stimuli. Experimental and control stimuli were balanced in terms of written frequency (67.2 vs. 67.3, respectively; *t* < |1|) and in terms of letter length (8.5 vs. 8.5; *t* < |1|).

(i) Words denoting proprioceptive states were selected starting from the examples considered in the studies of Berthoz (2000) and Craig (2003, 2009, 2010). Some examples are ‘agitation,’ ‘balance,’ ‘blush,’ ‘cramp,’ ‘spasm,’ ‘pain,’ ‘hunger,’ ‘cold,’ ‘fullness,’ ‘fever,’ ‘malaise,’ ‘relaxation,’ and ‘wheeze.’ (ii) The group of emotion words includes both transient emotions and moods. The list was freely composed starting from the examples given in a number of salient studies on the matter. We included only emotions that are considered to be culturally stable (more basic; Tomkins, 1962, 1963; Plutchik, 1980; Ekman, 1984, 1994, 1999; Reizenzein, 2009; Kassam et al., 2013). As for moods, we composed our list starting from the cases discussed by Ekman (1994), Damasio (1999), and Prinz (2004). Among the words we used were, e.g., ‘anguish,’ ‘amazement,’ ‘joy,’ ‘happiness,’ ‘sadness,’ ‘unhappiness,’ ‘disappointment,’ ‘love,’ ‘fear,’ ‘anger,’ ‘depression,’ ‘boredom,’ ‘distress,’ ‘indolence,’ and ‘relief.’ (iii) For the list of theoretical words we could not rely on previous databases of abstract words. In fact, as we already mentioned, theoretical words are not just abstract words, since many words which are considered abstract (not directly related to external perception), are indeed linked to internal – emotional or proprioceptive – sensory information (e.g., ‘friendship,’ ‘love,’ ‘freedom,’ or ‘wellness’ etc.; on this see also Vigliocco et al., 2009; Kousta et al., 2011). Thus, the class of *theoretical* words was defined *a priori*, with the intent of individuating a set of terms which rely primarily on verbal definitions like ‘axiom,’ ‘fallacy,’ hypothesis,’ ‘fraud,’ ‘democracy,’ ‘definition,’ ‘exception,’ ‘unanimity,’ ‘protocol,’ etc. (iv) Finally, we created a control set (matched for length and frequency with the others) composed uniquely of concrete words, i.e., of words which strongly rely on external sensory information. We included in this class terms like ‘table,’ ‘fennel,’ ‘candle,’ ‘juice,’ ‘cork,’ ‘pillow,’ ‘melon,’ ‘book,’ ‘stem glass,’ and ‘airplane.’

#### Questionnaires

##### Autistic traits (AQ)

Participants were administered the full 50-item Autism Quotient questionnaire (Baron-Cohen et al., 2001). Answering each question on the survey was mandatory, so there were no missing data for any participants who completed it. The results were scored according to Baron-Cohen et al.’s (2001) criteria, resulting in an “AQ score” for each participant. The AQ score represents the level of autistic traits/characteristics that participants possess.

##### Empathy traits (EQ)

Participants answered the 40-item short version of the Empathy Quotient questionnaire (Baron-Cohen and Wheelwright, 2004). The results were scored to obtain an “EQ score” for each participant, which represents their level of empathy traits, i.e., the ability to understand others’ emotions and moods.

#### Procedure

The survey was created and run between July 2014 and November 2014 using Google Forms Online Surveys.

Participants were asked to fill in two questionnaires (AQ, and EQ, Baron-Cohen et al., 2001; Baron-Cohen and Wheelwright, 2004) and to rate the imageability of a set of 360 words. Imageability was rated for each word on a 7-point Likert-like scale (one is the low and seven is the high imagery end of the scale). Instructions to participants were the Italian translation of Paivio et al.’s (1968) original instructions: “Nouns differ in their capacity to arouse mental images of things or events. Some words arouse a sensory experience, such as a mental picture or sound, very quickly and easily, whereas others may do so only with difficulty (i.e., after a long delay) or not at all. The purpose of this experiment is to rate a list of words as to the ease or difficulty with which they arouse mental images. Any word which, in your estimation, arouses a mental image (i.e., a mental picture, or sound, or other sensory experience) very quickly and easily should be given a high imagery rating: any word that arouses a mental image with difficulty or not at all should be given a low imagery rating. Think of the words ‘apple’ or ‘fact.’ Apple would probably arouse an image relatively easily and would be rated as high imagery; fact would probably do so with difficulty and would be rated as low imagery” (Paivio et al., 1968, p. 4; for an analysis of these instructions and of how people might plausibly interpret them to assign their ratings, see Pastore et al., 2015).

The experiment was conducted in two parts, each involving completion of an online form. In the first part, participants dealt with the form containing the AQ and a random selection of 180 words; in the second part, they dealt with the EQ and the remaining 180 words. Only the data from participants that completed all the four tasks (AQ, EQ, rating of word set 1 and word set 2) were used for the analyses.

#### Analytic Plan

First, for the whole group, we calculated correlations of AQ scores and EQ scores with the mean imageability ratings. We then determined correlations of AQ scores and EQ scores with the mean of the judgments of imageability for each of the word clusters (theoretical, emotional, proprioceptive, and control) for male and female participants. Statistical significance was set at *p* < 0.01.

Finally, imageability ratings were submitted to recursive partitioning (also known as tree-based models; Costello et al., 2003) to analyze how either the words’ category (theoretical, emotional, proprioceptive, control) or the single words predict the AQ and EQ scores.

## Results

### Preliminary Analysis

The data were explored and analyzed using R-project (version 3.1.1). Prior to data analysis, univariate and multivariate distributions of AQ, EQ and imageability judgment scores were examined for normality, homogeneity of variance, outliers, and influential cases (Fox, 1997). All these variables were normally distributed. The distance of each case to the center was evaluated to screen for multidimensional outliers (Fox, 1997). Autism Quotient scores and Empathy Quotient scores were, as expected, negatively correlated, *r* = -0.85, *p* < 0.001 sharing 72.3% of their common variance.

No significant differences emerged between female and male participants for the imageability judgment scores (*F* range = 0.01–1.8; ns). However, significant differences emerged between male and female for AQ [male *M* = 27.4; *SD* = 11.9; female *M* = 22.7; *SD* = 11.8; *F*(1,169) = 6.4; *p* < 0.05] and for EQ [male *M* = 30.5; *SD* = 14.3; female *M* = 38.6; *SD* = 16.0; *F*(1,169) = 11.2; *p* < 0.001].

### Correlational Analysis

For the whole group, neither AQ nor EQ were significantly correlated with mean imageability ratings (respectively, *r* = 0.07, ns and *r* = -0.08, ns).

**FIGURE 1:**
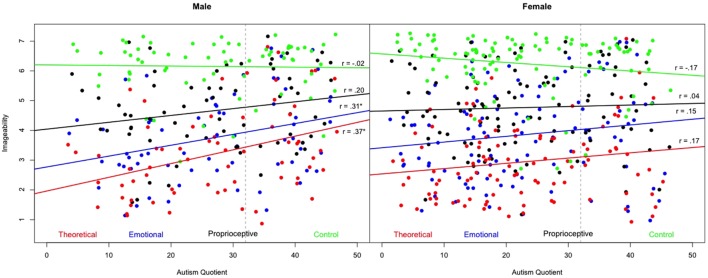
**Distributions of the Autism Spectrum Quotient (AQ) score and imageability ratings for each of the words clusters (theoretical, emotional, proprioceptive, and control) for male and female participants**.

**Figure 1** shows the linear correlations among the AQ scores and imageability ratings for each of the word clusters (theoretical, emotional, proprioceptive, and control) for male and female participants. There was a significant positive correlation for males (but not for females) between AQ and the imageability ratings of theoretical words (*r* = 0.37, *p* < 0.01), with the explained variance as low as 13.7%. There was a significant positive correlation for males (but not for females) between AQ and the imageability ratings of emotional words (*r* = 0.31, *p* < 0.01), with the explained variance as low as 9.6%. AQ was not significantly correlated for either males or for females with the imageability ratings of either proprioceptive words (male *r* = 0.20, ns; female *r* = 0.04, ns), or control words (male *r* = 0.02, ns; female *r* = -0.17, ns).

**FIGURE 2:**
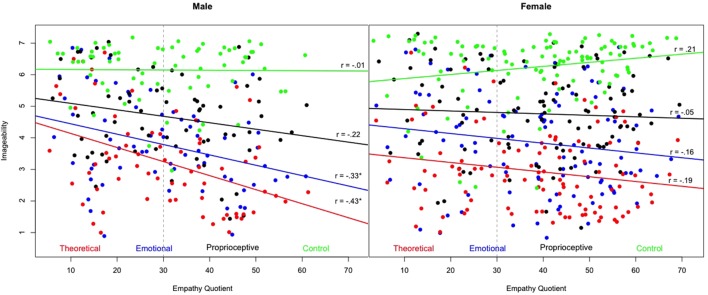
**Distributions of the Empathy Quotient (EQ) score and imageability ratings for each of the words clusters (theoretical, emotional, proprioceptive, and control) for male and female participants**.

**Figure 2** shows the linear correlations among the EQ scores and the imageability ratings for each of the word clusters (theoretical, emotional, proprioceptive, and control) for male and female participants. There was a significant negative correlation for males (but not for females) between EQ and the imageability ratings of theoretical words (*r* = -0.43, *p* < 0.01), with the explained variance as low as 18.5%. There was a significant negative correlation for males (but not for females) between EQ and the imageability ratings of emotional words (*r* = -0.33, *p* < 0.01), with the explained variance as low as 10.9%. EQ was not significantly correlated for either males or for females with the imageability ratings of either proprioceptive words (male *r* = -0.22, ns; female *r* = -0.05, ns), or control words (male *r* = -0.01, ns; female *r* = 0.21, ns).

### Tree-Base Model Analysis

#### Category-Words Level

**FIGURE 3:**
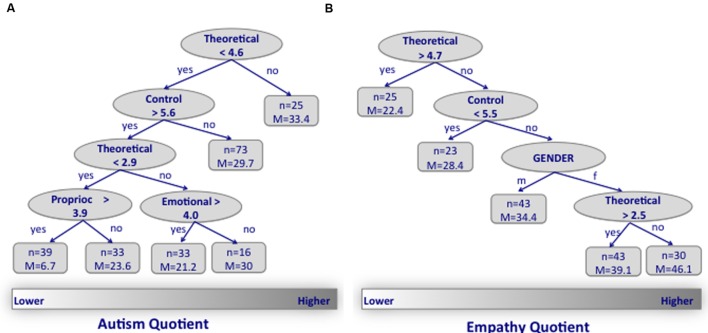
**(A)** The optimal tree that describes how imageability ratings of words’ category (theoretical, emotional, proprioceptive, and control) predict the AQ. The regression tree or tree-based model provides information about (1) the hierarchy of the importance of independent variables in explaining the distribution of the dependent variable and (2) which value of the independent variable divides the dependent variable in two groups that differ statistically. The bottom rectangle shows the distribution of expressed distress from lower (left) to higher (right). The values in the oval leaves of the tree refer to the condition of the independent variable that statistically divides the distribution of the dependent variable. Below each oval leaf, the indications “yes” or “no” refer to whether or not the condition is met. For the categorical variable “Gender” m, male and f, female. Each leaf is divided in two sub-leaves. The terminal leaves (quadrangles) represent subgroups that cannot be further subdivided. The *n*-value in the terminal leaves represents the size of the group, and *M* is the mean value of the group for the dependent variable. **(B)** The optimal tree for EQ.

**Figure 3** shows the optimal tree that describes how imageability ratings of the word categories (theoretical, emotional, proprioceptive, and control) predict the AQ (**Figure 3A**) and EQ (**Figure 3B**) scores. For both AQ and EQ scores, the judgment of *theoretical* words was the best measure that statistically differentiated the distribution. Specifically, participants with higher AQ (and conversely lower EQ) scores gave higher imageability ratings of the theoretical words (>4.6) than those with lower AQ (and higher EQ) scores. Next, the imageability ratings of the control and the theoretical words hierarchically stratified the AQ and EQ distributions, respectively. Finally, only for those participants with lower AQ (<30), the imageability ratings of the emotional and proprioceptive words affected the stratification of the population. Gender hierarchically stratified the distribution only for the EQ scores, with females having higher scores.

#### Single-Word Level

**FIGURE 4:**
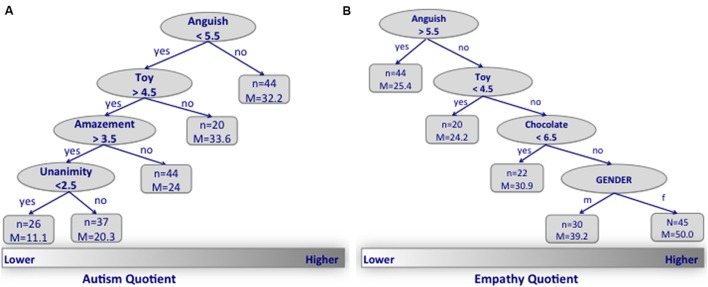
**(A)** The optimal tree that describes how imageability ratings of single words’ predict the AQ. **(B)** The optimal tree for EQ.

**Figure 4** shows the optimal tree which describes how imageability ratings at the single word level (all 360 words were considered independently) predicts the AQ (**Figure 4A**) and EQ (**Figure 4B**) scores. For both AQ and EQ scores, the ratings of the single word *anguish* were the best measure that statistically differentiated the distribution. Specifically, participants with higher AQ (and conversely lower EQ) scores gave lower (<5.55) imageability ratings to the word *anguish* than those with lower AQ (higher EQ) scores. Next for the AQ scores, the imageability judgments of the words *toy* (>4.5), *amazement* (<3.5), and *unanimity* (<2.5), hierarchically stratified the population. For the EQ scores, the imageability judgments of the words *toy* (>4.5) and *chocolate candy* (>6.5) hierarchically stratified the population. Gender hierarchically stratified the distribution only for the EQ scores, with females having higher scores.

## Discussion

The notion of mental imagery describes both the capacity to dynamically form and mentally recall representations of past sensory experience and the quasi-perceptual representations evoked by this process. Since they are private and subjective experiences, mental images are particularly difficult to investigate scientifically, even though they are crucial for understanding non-verbal thought and the relationship between verbal and non-verbal thought. This study offers a contribution to the comprehension of this phenomenon. Indeed, it allows us to establish a link between people’s lower vs. higher autistic traits (which we know to be related to a higher/lower ability to feel their own and others’ internal states, respectively) and their capacity to imagine specific word classes, i.e., to link specific words with quasi-perceptual information related to them. As has been reported in other studies on cognitive style in Autism (Kana et al., 2006; Mottron et al., 2006), this group probably compensates for the lack of direct emotional experience by developing a higher capacity to imagine emotions in a visual manner. This same tendency – even if to a lesser and non-significant degree – is discernible also in the case of proprioceptive terms referring to bodily (proprioceptive and interoceptive) states.

Conversely, we were also able to ascertain that people with high levels of autistic traits and lower levels of empathic traits have a more developed capacity to imagine theoretical words and this is compatible with the common knowledge that they are outstanding in understanding theoretical issues and in accomplishing abstract tasks. Thus, we can conclude that the capacity to dynamically form and mentally recall representations of past sensory experience associated with a word is in general positively related with a better grasp of the phenomena denoted by the words.

Furthermore, this study may also be relevant from a clinical perspective since the imageability ratings of word classes denoting proprioceptive, emotional and theoretical words allow us to predict whether people have low or high autistic traits, or, to put it differently, whether they have more or less marked empathic inclinations. In the DSM-5 it is at last acknowledged that people with autistic traits tend to belong to a spectrum. However, there are no specific markers that can easily predict where in the spectrum a person falls. Our study shows that a simple task such as rating mental imagery may be useful for performing a stratification of the population.

The effects we found are higher for the male than for the female participants. This is consistent with the so-called “extreme male brain theory of autism” and with the finding that autistic traits in men are generally more severe than those in women (Baron-Cohen, 2002).

Some limitations of this study point to future directions of study. Firstly, we can imagine that cognitive level may be a strong predictor of word imageability, so it is a limitation that no measures of cognitive performance were taken into account. However, in the current study we decided not to collect any IQ measure for technical reasons. Specifically, our procedure was very long (∼2 h in two sessions) and many participants had to be excluded because they did not finish the experiment. Adding another IQ test would have made our experiment even longer. However, we strongly believe that a follow-up study should include measures of the cognitive performance of the participants. Secondly, we tested our sample on ratings for a large number of words, and the task was long and demanding. To have clinical value, we believe the stratification of the population should be done based on the imagery rating of a smaller number of words. This will be possible by selecting only a subgroup of the words we have used, more specifically those words that lie at the extremes of the continuum. Furthermore, to evaluate possible clinical insights and applications, it would be useful to use this methodology with children. Indeed, conducting assessments at various ages during childhood could allow us to define the ontogeny of the imagery capacity across development and thus might be helpful in trying to identify atypical developmental trajectories. Finally, our research could be tested at the neurophysiological level in order to both identify brain neural networks that render the mental imagination of one class of words differently than another and to determine how they vary in different clinical groups.

## Conclusion

We used Paivio’s imagery scale to investigate whether autistic traits correlate with the capacity to imagine different kinds of words. The outcome is even more intriguing than we anticipated since it indicates that the ratings assigned by people to certain world classes can predict that person’s inclination to manifest autistic traits, including whether they fall above or below the diagnostic threshold.

People with more autistic traits have a higher capacity than typical matching controls to imagine theoretical, emotional and proprioceptive words. This may support the claim that people with high functioning autism compensate for their difficulties in understanding their internal states by using specific cognitive strategies related to imagery, more specifically by using associations to words referring to external sensory information. This finding certainly invites further investigation since it could lead to important insights for the development of psychological treatments. Furthermore, our results also suggest something more specific, i.e., that the reason people with high functioning autism show increased skills for theoretical tasks is not due to a highly developed capacity to recall verbal definitions or to build meaningful links among words; instead, these increased skills result from a capacity to form perceptual images of theoretical concepts.

## Author Contributions

All authors listed have made substantial, direct and intellectual contribution to the work, and approved it for publication.

## Conflict of Interest Statement

The authors declare that the research was conducted in the absence of any commercial or financial relationships that could be construed as a potential conflict of interest.

## Footnotes

1 Because ‘proprioception’ can be used in a wide sense to include also interoception, in the following we will speak in general of proprioceptive states/words.

## References

[B1] Acerra N. E. Moseley G. L. (2005). Dysynchiria: watching the mirror image of the unaffected limb elicits pain on the affected side. *Neurology* 65 751–753. 10.1212/01.wnl.0000178745.11996.8c 16157911

[B2] Anema H. A. Dijkerman H. C. (2013). “Motor and kinesthetic imagery,” in *Multisensory Imagery*, eds Lacey S. Lawson R. (New York, NY: Springer).

[B3] Baron-Cohen S. (2002). The extreme male brain theory of autism. *Trends Cogn. Sci.* 6 248–254. 10.1016/S1364-6613(02)01904-6 12039606

[B4] Baron-Cohen S. Wheelwright S. (2004). The empathy quotient: an investigation of adults with Asperger syndrome or high functioning autism, and normal sex differences. *J. Autism Dev. Dis.* 34 163–175. 10.1023/B:JADD.0000022607.19833.00 15162935

[B5] Baron-Cohen S. Wheelwright S. Skinner R. Martin J. Clubley E. (2001). The autism-spectrum quotient (AQ): evidence from asperger syndrome/high-functioning autism, males and females, scientists and mathematicians. *J. Autism Dev. Dis.* 31 5–17. 10.1023/A:1005653411471 11439754

[B6] Barsalou L. W. (1999). Perceptual symbol systems. *Behav. Brain Sci.* 22 577–660. 11301525 10.1017/s0140525x99002149

[B7] Barsalou L. W. (2008). Grounded cognition. *Annu. Rev. Psychol.* 59 617–645. 10.1146/annurev.psych.59.103006.093639 17705682

[B8] Barsalou L. W. Niedenthal P. M. Barbey A. K. Ruppert J. A. (2003). “Social embodiment,” in *The Psychology of Learning and Motivation. Advances in Research and Theory* Vol. 43 ed. Ross B. H. (Amsterdam: Academic Press).

[B9] Barsalou L. W. Wiemer-Hastings K. (2005). “Situating abstract concepts,” in *Grounding Cognition: The Role of Perception and Action in Memory, Language, and Thought*, eds Pecher D. Zwaan R. (New York, NY: Cambridge University Press), 129–163.

[B10] Berthoz A. (2000). *The Brain’s Sense of Movement*. Cambridge: Harvard University Press.

[B11] Coltheart M. (1981). The MRC psycholinguistic database. *Q. J. Exp. Psychol. A Hum. Exp. Psychol.* 33 497–505. 10.1080/14640748108400805

[B12] Connell L. Lynott D. (2012). Strength of perceptual experience predicts word processing performance better than concreteness or imageability. *Cognition* 125 452–465. 10.1016/j.cognition.2012.07.010 22935248

[B13] Costello T. J. Swartz M. D. Sabripour M. Gu X. Sharma R. Etzel C. J. (2003). Use of tree-based models to identify subgroups and increase power to detect linkage to cardiovascular disease traits. *BMC Genet.* 4:S66 10.1186/1471-2156-4-S1-S66 PMC1866504 14975134

[B14] Craig A. D. (2003). Interoception: the sense of the physiological condition of the body. *Curr. Opin. Neurobiol.* 13 500–505. 10.1016/S0959-4388(03)00090-4 12965300

[B15] Craig A. D. (2009). How do you feel – now? The anterior insula and human awareness. *Nat. Rev. Neurosci.* 10 59–70. 10.1038/nrn2555 19096369

[B16] Craig A. D. (2010). The sentient self. *Brain Struct. Funct.* 214 563–577. 10.1007/s00429-010-0248-y 20512381

[B17] Dalsant A. Truzzi A. Setoh P. Esposito G. (2015). Empatia e Teoria della Mente: un unico meccanismo cognitivo [Empathy and theory of mind: one underlying cognitive mechanism]. *Riv Int. Filos. Psicol.* 6 245–248.

[B18] Damasio A. (1999). *The Feeling of What Happens. Body and Emotion in Making of Consciousness.* New York, NY: Mariner Books.

[B19] Dellantonio S. Job R. Mulatti C. (2014a). Imageability: now you see it again (albeit in a different form). *Front. Psychol.* 5:279 10.3389/fpsyg.2014.00279 PMC3982064 24765083

[B20] Dellantonio S. Mulatti C. Pastore L. Job R. (2014b). Measuring inconsistencies can lead you forward: imageability and the x-ceptive theory. *Front. Psychol.* 5:708 10.3389/fpsyg.2014.00708 PMC4097956 25076920

[B21] Ekman P. (1984). “Expression and the nature of emotion,” in *Approaches to Emotion*, eds Schererand K. Ekman P. (Hillsdale, NJ: Lawrence Erlbaum), 319–343.

[B22] Ekman P. (1994). “Moods, emotions and traits,” in *The Nature of Emotion: Fundamental Questions*, eds Ekman P. Davidson R. J. (Oxford: Oxford University Press), 56–58.

[B23] Ekman P. (1999). “Basic emotions,” in *Handbook of Cognition and Emotion*, eds Dalgleish T. Power M. (Sussex, NJ: John Wiley & Sons), 45–60.

[B24] Fox J. (1997). *Applied Regression Analysis, Linear Models and Related Methods*. Thousand Oaks, CA: Sage.

[B25] Gaigg S. B. (2012). The interplay between emotion and cognition in autism spectrum disorder: implications for developmental theory. *Front. Integr. Neurosci.* 6:113 10.3389/fnint.2012.00113 PMC3540960 23316143

[B26] Gentili C. Cristea G. A. Ricciardi E. Costescu C. David D. Pietrini P. (2015). Neurobiological Correlates of the Attitude Toward Human Empathy. *Riv. Int. Filos. Psicol.* 6 70–87. 10.4453/rifp.2015.0006

[B27] Gollnisch G. Averill J. R. (1993). Emotional imagery: strategies and correlates. *Cogn. Emot.* 7 407–429. 10.1080/02699939308409196

[B28] Kana R. K. Keller T. A. Cherkassky V. L. Minshew N. J. Just M. A. (2006). Sentence comprehension in autism: thinking in pictures with decreased functional connectivity. *Brain* 129 2484–2493. 10.1093/brain/awl164 16835247 PMC4500127

[B29] Kassam K. S. Markey A. R. Cherkassy V. L. Loewenstein G. Just M. A. (2013). Identifying emotions on the basis of neural activation. *PLoS ONE* 8:e66032 10.1371/journal.pone.0066032 PMC3686858 23840392

[B30] Kousta S. Vigliocco G. Vinson D. Andrew A. Del Campo E. (2011). The representation of abstract words: why emotion matters. *J. Exp. Psychol. Gen.* 140 14–34. 10.1037/a0021446 21171803

[B31] Matsuda S. Yamamoto J. (2015). Intramodal and cross-modal matching of emotional expression in young children with autism spectrum disorders. *Res. Autism Spectr. Disord.* 10 109–115. 10.1016/j.rasd.2014.11.010

[B32] Mottron L. Dawson M. Soulieres I. Hubert B. Burack J. (2006). Enhanced perceptual functioning in autism: an update, and eight principles of autistic perception. *J. Autism Dev. Disord.* 36 27–43. 10.1007/s10803-005-0040-7 16453071

[B33] Paivio A. (1971). *Imagery and Verbal Processes.* New York, NY: Holt, Rinehart & Winston.

[B34] Paivio A. (1986). *Mental Representations. A Dual Coding Approach.* Oxford: Oxford University Press.

[B35] Paivio A. (1991). Dual coding theory: retrospect and current status. *Can. J. Psychol.* 45 255–287. 10.1037/h0084295

[B36] Paivio A. (2007). *Mind and Its Evolution: A Dual Coding Theoretical Approach*. Mahwah, NJ: Erlbaum.

[B37] Paivio A. Yuille J. C. Madigan S. A. (1968). Concreteness, imagery, and meaningfulness values for 925 nouns. *J. Exp. Psychol.* 76 1–25. 10.1037/h0025327 5672258

[B38] Pastore L. Dellantonio S. Mulatti C. Job R. (2015). “On the nature and composition of abstract (theoretical) concepts: the X-ception theory and methods for its assessment,” in *Philosophy and Cognitive Science II* Vol. 20 eds Magnani L. Ping L. Park W. (Heidelberg: Springer), 35–58. 10.1007/978-3-319-18479-1_3

[B39] Plutchik R. (1980). “A general psychoevolutionary theory of emotion,” in *Emotion: Theory, Research, and Experience: Theories of Emotion* Vol. 1 eds Plutchik R. Kellerman H. (New York, NY: Academic Press), 3–33.

[B40] Prinz J. (2002). *Furnishing the Mind: Concepts and Their Perceptual Basis*. Cambridge: MIT Press.

[B41] Prinz J. J. (2004). *Gut Reactions. A Perceptual Theory of Emotion.* Oxford: Oxford University Press.

[B42] Reizenzein R. (2009). Emotional experience in the computational belief-desire theory of emotion. *Emot. Rev.* 1 214–222. 10.1177/1754073909103589

[B43] Roeckelein J. (2004). *Imagery in Psychology: A Reference Guide*. Westport, CT: Praeger.

[B44] Ronald A. Happe F. Price T. S. Baron-Cohen S. Plomin R. (2006). Phenotypic and genetic overlap between autistic traits at the extremes of the general population. *J. Am. Acad. Child Adolesc. Psychiatry* 45 1206–1214. 10.1097/01.chi.0000230165.54117.41 17003666

[B45] Switras J. E. (1978). An alternate-form instrument to assess vividness and controllability of mental imagery in seven modalities. *Percept. Mot. Skills* 46 379–384. 10.2466/pms.1978.46.2.379 662535

[B46] Tomkins S. S. (1962). *Affect, Imagery, Consciousness: The Positive Affects*, Vol. I New York, NY: Springer.

[B47] Tomkins S. S. (1963). *Affect, Imagery, Consciousness: The Negative Affects*, Vol. II New York, NY: Springer.

[B48] Vigliocco G. Meteyard L. Andrews M. Kousta S. (2009). Toward a theory of semantic representation. *Lang. Cogn.* 1 219–247. 10.1515/LANGCOG.2009.011

[B49] Wilson M. D. (1988). The MRC psycholinguistic database: machine readable rictionary, version 2. *Behav. Res. Methods Instrum. Comput.* 20 6–11. 10.3758/BF03202594

